# Aging affects the biological activity of fibroblast growth factor (FGF) in gastric epithelial cell, which is partially rescued by uridine

**DOI:** 10.1080/21655979.2022.2029066

**Published:** 2022-02-01

**Authors:** Xiaomei Zhang, Huifeng Zhang, Jingli Gong, Huan Yu, Di Wu, Junyu Hou, Minghui Li, Xin Sun

**Affiliations:** a School of Pharmacy, Jilin Medical University, Jilin city, Jilin Province, 132013 China; b School of Pharmacy, Beihua University, Jilin City, China

**Keywords:** FGF, FGFR, aging, gastric epithelial cell, uridine

## Abstract

Aging has become an irreversible trend in the world, the health problems caused by aging cannot be ignored. The physiological functions of human body begin to decline with aging, the decline of gastrointestinal function caused by aging is an important problem that needs to be resolved. In this work, we evaluated the anti-aging effect of uridine in the senescent gastric epithelial cell model, and found that the aging level of gastric epithelial cell was significantly down-regulated by uridine treatment, uridine could obviously down-regulate the ratio of the SA-β-gal-positive senescent cells. Furthermore, aging-related marker molecules (such as p16 and p21) were also significantly down-regulated under uridine treatment. Additionally, the levels of inflammation and oxidative stress were also significantly reduced by uridine treatment. Next, our further studies the effect of aging on FGF activity on gastric epithelial cell, and found that FGF/FGFR-mediated signaling pathways were significantly down-regulated. However, uridine treatment can not only alleviate the senescence of gastric epithelial cell, but also can partially restore the sensitivity of FGF signaling. Taken together, the current work indicates that uridine shows a good anti-aging effect, which lays a solid foundation for the related research in this filed.

## Graphical abstract

**Figure:**
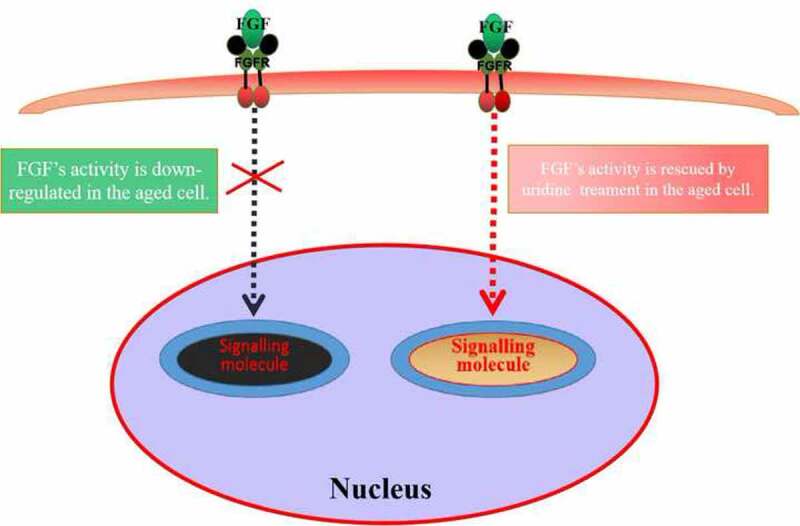

## Introduction

Fibroblast growth factor (FGF) is a growth factor, which was first found in brain tissue and pituitary extract [1]. FGFs are widely distributed in various tissues and organs [2]. FGFs exist mainly in two forms according to their isoelectric points: basic fibroblast growth factor (bFGF) and acidic fibroblast growth factor (aFGF). Their structures and functions are very similar. bFGF is involved in cell growth and differentiation, angiogenesis, embryonic tissue development, tissue and organ formation, and metabolism [1]. bFGF exerts its biological functions by binding to its specific receptors (FGFR) expressed on cell membrane. FGFR is a receptor tyrosine kinase with four key members (FGFR1 ~ 4). They share the same structural domain, including extracellular LG like domain and intracellular TK domain. The biological function of FGF/FGFR is very extensive. FGF could perform different biological functions in different physiological conditions and environments [3]. The main signal pathways mediated by FGFR include Ras-MAPK signal pathway, JAK/STAT signal pathway, and other signaling pathways, which cooperate to play the biological role and function of FGF.

With the advent of aging society, aging is an important scientific problem that human beings must be faced [4]. As the digestive system, the gastrointestinal tract can absorb nutrition and energy from food. In addition, the gastrointestinal tract also plays an important role in barrier function, which can protect organisms from the external environment damage. Clinical studies have reported that gastrointestinal motility is weakened with aging [5], which significantly reduces the quality of life of patients and brings a huge economic burden to the health care system. Previous studies found that FGF has many important biological effects on the stomach [6,7]. For example, FGF could stimulate gastric epithelial cell proliferation [8]. In addition, it has been reported that bFGFis involved in the damage repair of gastric mucosal cell [9].

FGF plays an important biological role in the proliferation of stomach, whether stomach aging affects the biological activity of FGF? If so, we should find a bioactive molecule with the potential of anti-aging of gastric cells. Therefore, in the current study, we also evaluate the biological activity of uridine on aging gastric epithelial cells. Uridine is a precursor of pyrimidine nucleotides which is necessary for RNA synthesis. It is a major nucleotide analogue in mammalian milk [10]. Uridine has many important biological effects. It has been reported that, uridine could reduce cytotoxicity [11] and improve neurophysiological function [12,13]. Additionally, uridine could regulate the intestinal health [10].

In the current study, we established the senescent cell model (gastric cell) by H_2_O_2_ treatment. Further research indicated that the biological activity of FGF was significantly reduced in the senescent GES-1 cell (human gastric epithelial cell line) and RGM-1 (gastric epithelial cell line). Next, we found that uridine has anti-aging effect and can partially restore the sensitivity of FGF in the senescent cell model. Taken together, the current work indicates that: 1) Uridine can alleviate the senescence of gastric epithelial cell; 2) the activities of FGF is reduced in the senescent gastric cell; 3) Uridine can partially restore the sensitivity of FGF in the senescent gastric cell.

### Materials and methods

#### Reagents and antibodies

RNA extraction kit was purchased form Invitrogen (USA). DMEM culture medium and Fetal bovine serum (FBS) were purchased from thermo fisher scientific. RIPA lysate, PVDF and bovine serum albumin (BSA) were obtained from Beyotime (Shanghai, China). MTT kit was obtained form Beyotime Biotechnology Co., Ltd (Shanghai, China). Anti-FGFR (#ab76464 at 1/100 dilution), anti-STAT3 (#ab68153 at 1/200 dilution) and aniti-STAT5 (#ab230670 at 1/500 dilution) were obtained from Abcam (UK). Anti-total AKT (#9272 at 1/200 dilution) and Anti-p-AKT (#5677 at 1/100 dilution), Anti-ERK1/2 (#4986 at 1/200 dilution) and Anti-p-ERK1/2 (#5554 at 1/200 dilution) were purchased from CST. Alexa-488 conjugated goat anti-mouse was obtained from Molecular Probes (Life Technologies, Sweden). All reagents were purchased from Sigma without special instructions.

### Cell culture

The GES-1 cells (human gastric epithelial cell) and RGM-1 (gastric epithelial cell) were stored in our laboratory. The GES-1 cell was cultured in DMEM medium supplemented with 10% serum and 100 units/mL penicillin and streptomycin at 37°C in a humidified incubator with 5% CO_2_. The rat gastric mucosal cell line (RGM-1) was grown in DMEM/F12 medium containing 10% FCS.

### Rt-PCR

Total RNA was extracted with the TRIzol reagent according to the manufacturer’s instructions. The quality and integrity of the extracted RNA were evaluated by using Agilent 2100 Bioanalyzer (Agilent,Santa Clara, CA, USA). The total RNA concentration was determined by nanodrop 2000 spectrophotometer (Thermo Scientific, Waltham, MA, USA). RNA was reverse-transcribed into cDNA according to manufacturer’s instructions. RT-PCR analyses were then performed.

### Western-blot

GES-1 and RGM-1 cell concentrations were adjusted to 1.0 × 10^6^/ml and seeded into the cell culture plate. After the cells grew to the logarithmic growth phase, they were stimulated with FGF (30 ng/mL). After FGF treatment, the culture medium containing FGF was absorbed. After washing, the cells were lysed, and then the cell lysate was centrifuged at 10,000 RPM/min for 10 min. The precipitate was discarded and the supernatant was collected. The cell concentration was measured by BCA method. The samples were separated by SDS-PAGE and transferred to PVDF membrane. After washing, the membrane was sealed with 5% BSA at 37°C for 2 h. After washing, the primary antibody was added and incubated for 12 h at 4°C. After washing, the secondary antibody was added and incubated for 2 h. After washing, the immunoprotein band was detected by ECL kit.

### Detection of cell cycle by flow cytometry

After the cells were stimulated by FGF (30 ng/mL), GES-1 and RGM-1 cells were cultured to logarithmic growth stage. The cells were then digested using trypsin (containing EGTA) at 37^°^C, and the digestion was terminated with DMEM medium containing FBS. The cells were then collected by centrifugation (300 g, 5 min). The cells were washed twice with PBS, and were fixed with 70% ethanol. The cells were collected by centrifugation (300 g, for 5 min). After the cells were washed, 100 μL RNAse solution was added and incubated at 37°C for 30 min, after which, 400 μL PI solution was added to stain the cells for 30 min.

### Flow cytometry analysis of intracellular signaling pathways

After GES-1 and RGM-1 cells were cultured to logarithmic growth stage, the cells were digested with trypsin (containing EGTA), and the digestion was terminated with medium containing FBS. The cells were collected by centrifugation (1000 rpm, 5 min). The cells were washed twice with PBS, the cells were then fixed with 70% ethanol. After the cells were blocked with 5% BSA, the primary antibody was added and incubated for 2 h at 37°C. After washing, fluorescent labeled secondary antibodies were added and incubated for 2 h, and then flow cytometry analysis was performed to evaluate the cell samples.

### Indirect immunofluorescence assay (IFA)

When the cells grew to about 30–40% confluence, the cells were washed twice using PBS. The cells were then fixed with 4% PFA at room temperature for 1 h. After the cells were blocked using BSA, the indicated primary antibody was added and incubated for 2 h at 37°C. After washing, fluorescent-labeled secondary antibodies were added and incubated for 2 h, and then observed by laser confocal microscope (Carl Zeiss).

### MTT assay

GES-1 and RGM-1 cells in logarithmic growth stage were digested and suspended in serum-free medium. Cell samples were distributed into a 96-well cell culture plate (2 × 10^4^/well), and incubated for 24 h. The GES-1 and RGM-1 cells were then challenged with FGF and cultured for 24–48 h. After washing with PBS, the MTT (50 μL/well) reagent was added into each well and incubated for 120 min. The optical density (OD) value in the each well was detected with a microplate reader (Bio-rad, imark).

### Apoptosis analysis by flow cytometry

The cell apoptosis was evaluated by Annexin V-Alexa Fluor488/PI Apoptosis Detection Kit according to manufacture’s instructions. In brief, GES-1 and RGM-1 cells were digested and collected by centrifugation (300 × g for 5 min). PBS solution was used to suspend cell pellets. After fixing the cells with 75% ethanol for 30 min at RT, PI solution (5 μg/ml) was then added and incubated for 30 min at RT. After washing the cells for three times, the cell samples were detected by Flow cytometry (FCM). CELLQUEST software (BD Biosciences) was used to analyze the data.

### ELISA assays

The levels of proinflammatory cytokines were detected using an ELISA kit according to the instructions of the manufacture. In brief, cellular proteins were extracted, and the intracellular protein samples were added into ELISA plate. The plates were washed for three times, and blocking solution (5%BSA) was then added and incubated for 1 h at 37°C, after which, the secondary antibody was added and incubated for 60 min. After three washes, TMB solution was added to each well. The optical density (OD) was detected at a wavelength of 450 using ELISA reader (Bio-rad).

### Statistical analysis

Statistical analysis was performed using Prism version 8.0 software (Graph Pad software). The data are expressed as mean ± SEM. Student’s t-test and One-way Analysis of Variance were used for comparison. p < 0.05 was defined as statistically significant.

## Results

In this work, we established the senescent cell model (gastric epithelial cell) by H_2_O_2_ treatment. Our work indicated that the biological activity of FGF was significantly reduced in the senescent GES-1 cell and RGM-1 cells. Next, we found that uridine has anti-aging effect and could partially rescue the sensitivity of FGF signaling in the senescent cell model. Taken together, the current work indicates that: 1) Uridine can alleviate the senescence of gastric epithelial cell; 2) The activities of FGF is reduced in the senescent gastric cell; 3) Uridine can partially rescue the sensitivity of FGF in the senescent gastric cell.

### Cell senescence models of GES-1 and RGM-1 was successfully constructed by H_2_O_2_ treatment

**Figure 1.**
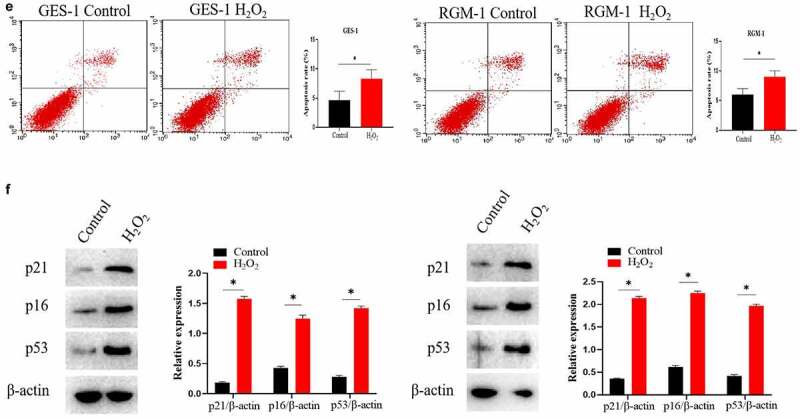
(a). Sa-β-gal positive cells significantly increased by H_2_O_2_ stimulation. The experiments were performed by β-Galactosidase Staining Kit (Solarbio, G8860) according to the manufacturer’s instructions. (b). Cell proliferation were reduced after H_2_O_2_ treatment by MTT assay. GES-1 and RGM-1 cells were exposed to H_2_O_2_ for 2 h. The cells were then distributed into a 96-well cell culture plate (2 × 10^4^/well), and incubated for 24 h. After washing with PBS, the MTT (50 μL/well) reagent was added into each well and incubated for 120 min. The optical density (OD) value in the each well was detected with a microplate reader (Bio-rad, imark). n = 3 biological replicates. (c). The ratio of S phase cells were significantly decreased by H_2_O_2_ treatment. n = 3 biological replicates. (d). The expressions of cyclin D1 and Ki67 were also significantly down-regulated by Western-blot analysis. E. Analysis of cell apoptosis by flow cytometry. F. P15 and P16 were significantly up-regulated by H_2_O_2_ treatment. The data are shown as means ± SEM. Asterisks indicate significant differences (P < 0.05).

In order to study the effect of gastric cell aging on bioactivity of bFGF, we constructed the cell senescence models. For this, GES-1 and RGM-1 cells were treated with various concentrations of H_2_O_2_ (0–100 μmol/L) for 120 min. After H_2_O_2_ treatment, the medium containing H_2_O_2_ was discarded and a fresh medium was added. The cells were cultured for another 24 h. The senescence of GES-1 and RGM-1 cells were then checked by evaluating the aging-related marker molecules. Sa-β-gal is a specific marker of aging. Therefore, we firstly analyzed the Sa-β-gal staining of GES-1 and RGM-1 cell. It can be seen that the H_2_O_2_ (50 μmol) leaded to GES-1 and RGM-1 cell senescence. Sa-β-gal staining showed that the proportion of Sa-β-gal positive cells significantly increased compared with the control group (Figure 1a). The MTT assays showed that cell proliferation was reduced after H_2_O_2_ treatment (Figure 1b). Additionally, the ratio of S phase cells decreased significantly (Figure 1c), and the expressions of cyclin D1 and Ki67 were also significantly down-regulated (Figure 1d). Flow cytometry evaluation showed that H_2_O_2_ (50 μmol) slightly induced the cell apoptosis (GES-1 and RGM-1) (Figure 1e). Additionally, we further checked the expression of cell senescence markers, it can be seen that the relative expression level of P15 and P16 were significantly up-regulated compared to control group (Figure 1f).**Figure:**
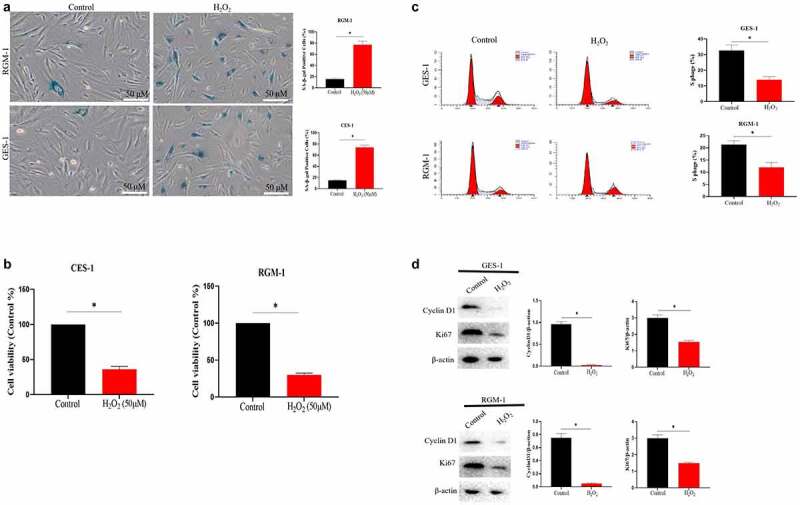

### FGF’s intracellular trafficking in normal and senescent GES-1 and RGM-1 cells

**Figure 2.**
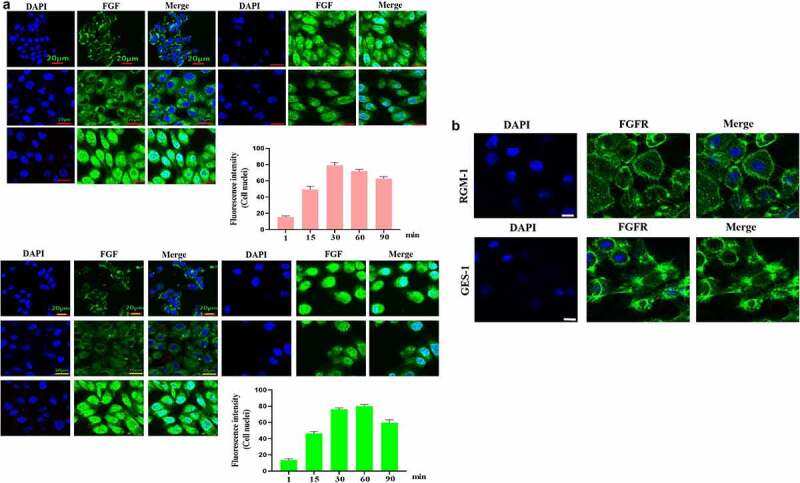
(a). The GES-1 and RGM-1 cells were seeded on 6-well cell culture plates. 40% confluent cells were starved for 10 h. The cells were then stimulated with FITC-FGF for the indicated time points. After the cells were fixed, the cells were observed using CLSM. (b). FGF could not transport into the nucleus in the senescent cells. The average fluorescence intensity of 50 cells was measured and analyzed. Asterisks indicate significant differences (P < 0.05).

In the normal GES-1 and RGM-1 cell, we checked the intracellular trafficking of FGF by CLSM. We can see that FGF internalized into the GES-1 and RGM-1 cells in a time-course dependent manner. After FGF exposure for 1 min, FITC-labeled bFGF was mainly localized on the cell membrane of GES-1 and RGM-1. With the extension of FITC-FGF incubation time (15–30 min), and the fluorescent signal in the cell was constantly enhanced. After FGF exposure for 45–60 min, the FGF fluorescence signal in the cell reached the peak, and then began to decline (Figure 2).

It is interesting that we found that FGF also transported into the cell nuclei of GES-1 and RGM-1 cells. However, in the senescent GES-1, we can see that although FGF could still internalize into the cell, FGF could not transport into the nucleus (Figure 2b).

### FGF-induced FGFR’s intracellular trafficking in normal and senescent GES-1 and RGM-1 cells

**Figure 3.**
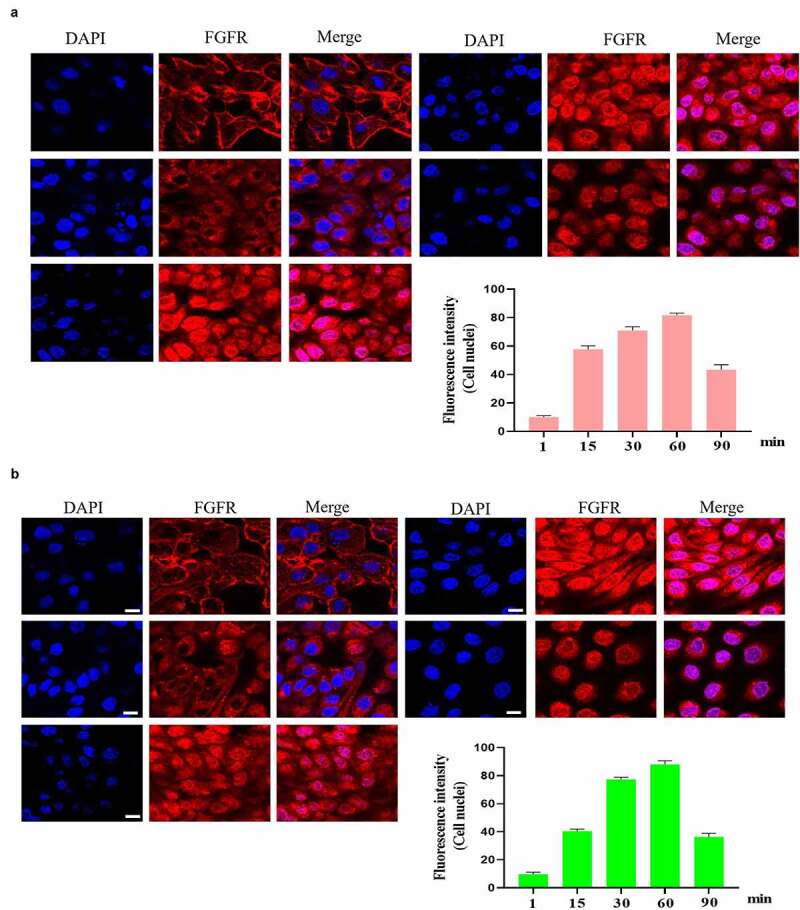
(a-b). FGFR’s intracellular trafficking under FGF stimulation. The GES-1 and RGM-1 cells were seeded on 6-well cell culture plates. 40% confluent cells were starved for 10 h. The cells were then stimulated with FGF for the indicated time points. After the cells were fixed, the cells were stained with anti-FGFR. The cell samples were then observed using CLSM. The average fluorescence intensity of 50 cells was measured and analyzed. Asterisks indicate significant differences (P < 0.05).

In the normal GES-1 and RGM-1 cells, the intracellular trafficking of FGFR induced by FGF was evaluated by CLSM using anti-FGFR antibody (#ab76464 at 1/100 dilution). CLSM analyses indicated that FGFR transported into the GES-1 and RGM-1 cells in a time-dependant manner. After FGF treatment for 1 min, FGFR was detected mainly on the cell membrane of GES-1 and RGM-1 (Figure 3a–b). With the extension of FGF treatment time (15–30 min), and The amount of fluorescence signal (FGFR) was increased in cells. After FGF exposure for 45–60 min, the fluorescence signal of FGFR in the cell reached the maximum, and then began to decline.

**Figure 4.**
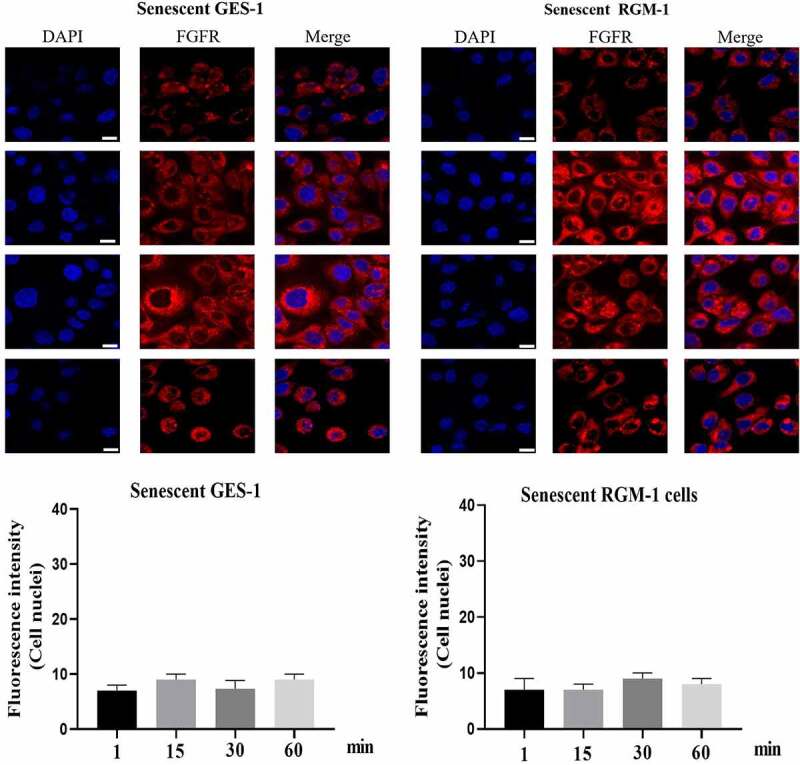
(a). FGFR’s intracellular trafficking under FGF stimulation in senescent GES-1 and RGM-1 cells. The GES-1 and RGM-1 cells were seeded on 6-well cell culture plates. 30–50% confluent cells were starved for 10 h. The cells were then stimulated with FGF for the indicated time points. After the cells were fixed, the cells were stained with anti-FGFR. The cell samples were then observed using CLSM. The average fluorescence intensity of 50 cells was measured and analyzed. Asterisks indicate significant differences (P < 0.05).

It is interesting that FGFR translocated into the cell nuclei of GES-1 and RGM-1 cells. However, in the senescent GES-1 and RGM-1, FGFR can not transport into the nucleus (although FGFR could internalize into the cell) (Figure 4).

**Figure 5.**
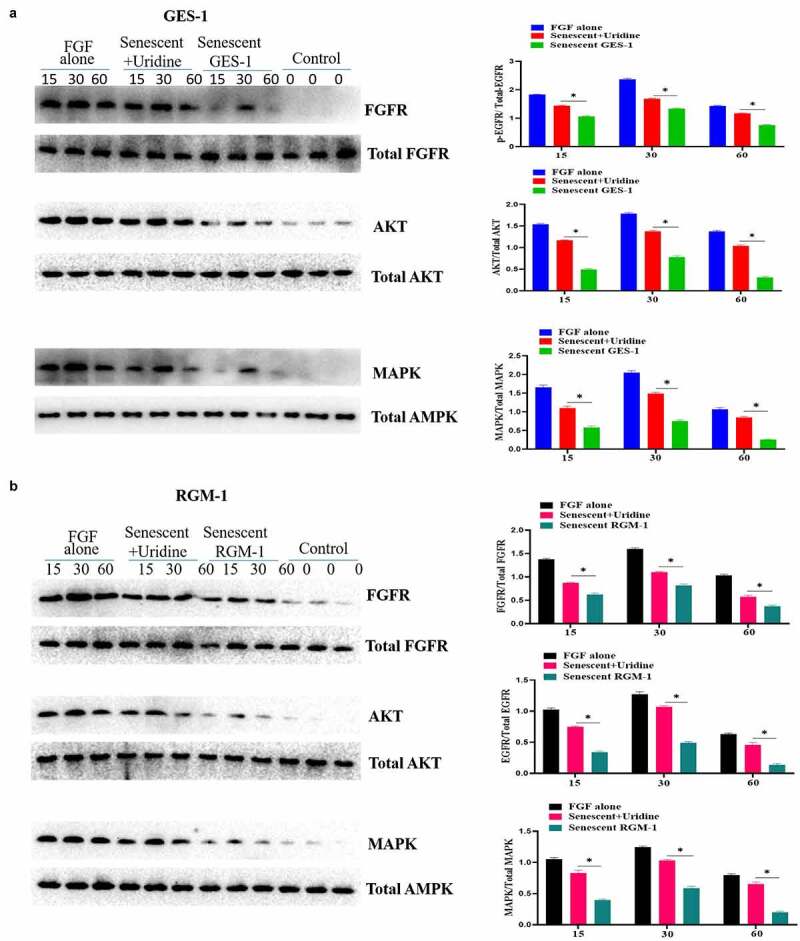
Uridine treatment partially restored the signaling ability of FGF. GES-1 and RGM-1 cell were stimulated with FGF (30 ng/ml) for the indicated time point. Then, the cell lysate was used to lyse the cells, and then the cell lysate was centrifuged at 10,000 RPM/min for 10 min. the precipitate was discarded and the supernatant was collected. The cell concentration was measured by BCA method. The samples were separated by SDS-PAGE and transferred to PVDF membrane. After washing twice, the membrane was sealed with 5% BSA at 37°C for 2 h. After washing, the primary antibody was added and incubated for 12 h at 4°C. After washing, the secondary antibody was added and incubated for 2 h. After washing, the immunoprotein bands were detected by ECL kit.

The effect of aging on FGF signaling was evaluated in the senescent GES-1 and RGM-1, it can be found that the FGFR-mediated intracellular signaling was significantly suppressed when compared to control group. Figure 5 A and B indicated that the phosphorylation levels of FGFR-mediated signaling proteins were down-regulated in the senescent cells. These results indicated that FGFR-mediated signaling sensitivity was significantly down-regulated in the senescent GES-1 and RGM-1 cell model. It is interesting that Uridine treatment partially restored the FGFR-mediated introcellular signaling.

### Study of the mechanism by which the FGFR-mediated intracellular signaling was inhibited in the senescent GES-1 and RGM-1 cells

**Figure 6.**
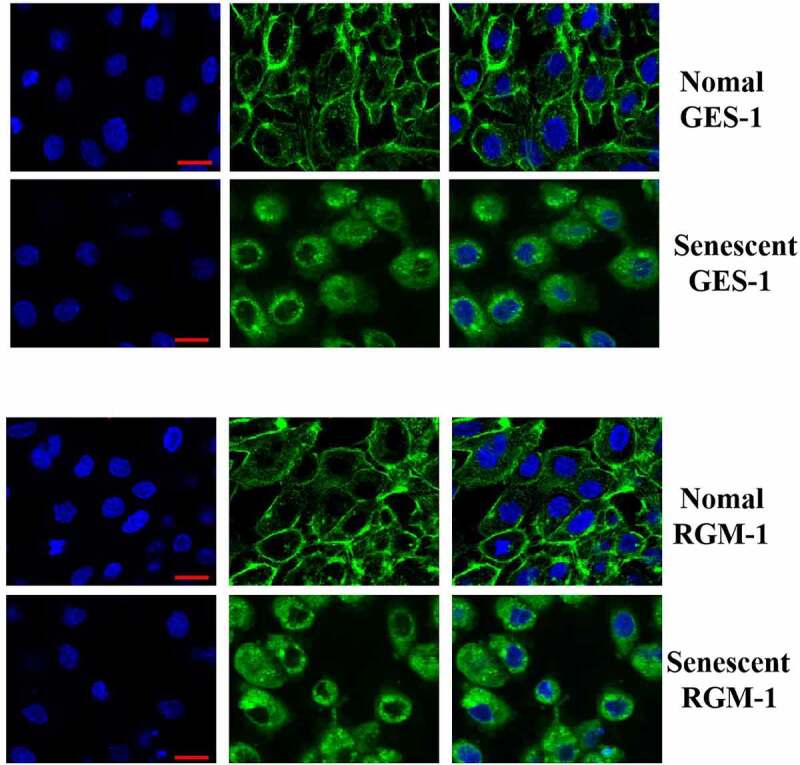
The GES-1 and RGM-1 cells were seeded on cover slip in 6-well cell culture plates. The cells were then fixed by 4% PFA at RT for 1 h. After blocking with 3% BSA, the anti-FGFR (1:1000) were added and incubated for 2 h at RT. After washing, fluorescent labeled secondary antibodies were added and incubated for 2 h, and then the cell samples were observed by CLSM. Asterisks indicate significant differences (P < 0.05).

We further explored the potential mechanism by which FGF-mediated signaling was down-regulated in the senescent cells. Firstly, we detected the total FGFR expression by western-blot analysis, and we didnot detect a significant difference in total FGFR expression. Next, the FGFR’s expression was evaluated by CLSM, we found an interesting phenomenon, the expression of membrane-localized FGFR was significantly down-regulated in the senescent cells, but the expression of cytoplasmic FGFR was significantly increased, which may be one of the molecular mechanisms of FGF signaling down-regulation (Figure 6).

### Uridine shows anti-aging effects in the senescent GES-1 and RGM-1

**Figure 7.**
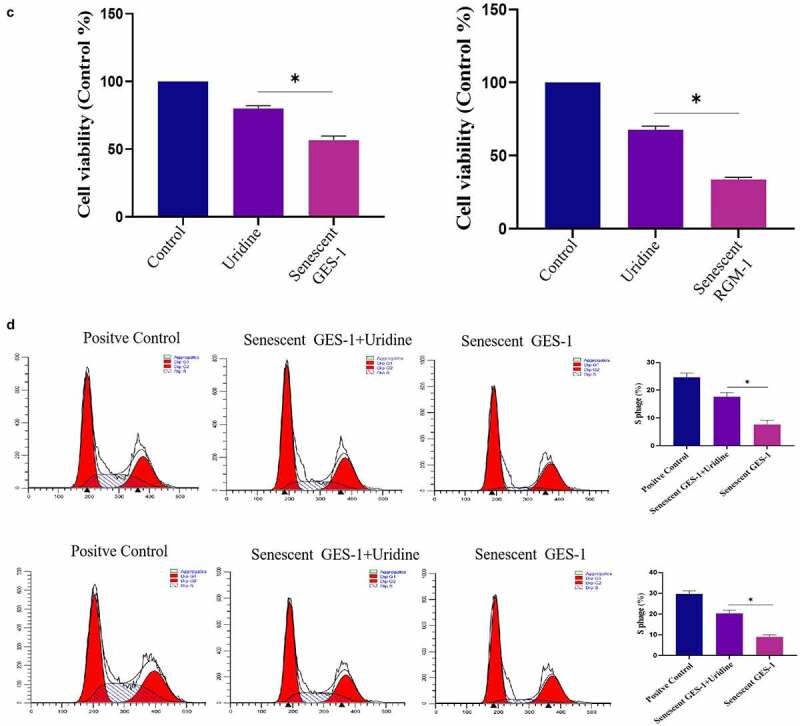
(Continued)

The effect of uridine on the senescent GES-1 and RGM-1 were evaluated. Figure 7a showed that uridine (0.4 mg) treatment significantly alleviated the senescence of GES-1 and RGM-1 cells, the senescence-associated β-galactosidase (SA β-gal)-positive cell ratio was significantly lower than that of control group. Furthermore, the expression levels of senescence markers (p16, p21 and p53) were down-regulated by uridine treatment (Figure 7b). MTT analyses showed that the uridine treatment increased cell proliferation ability of the senescent GES-1 and RGM-1 cell (Figure 7c). Additionally, uridine treatment changed cell cycle (Figure 7d).**Figure 7.**
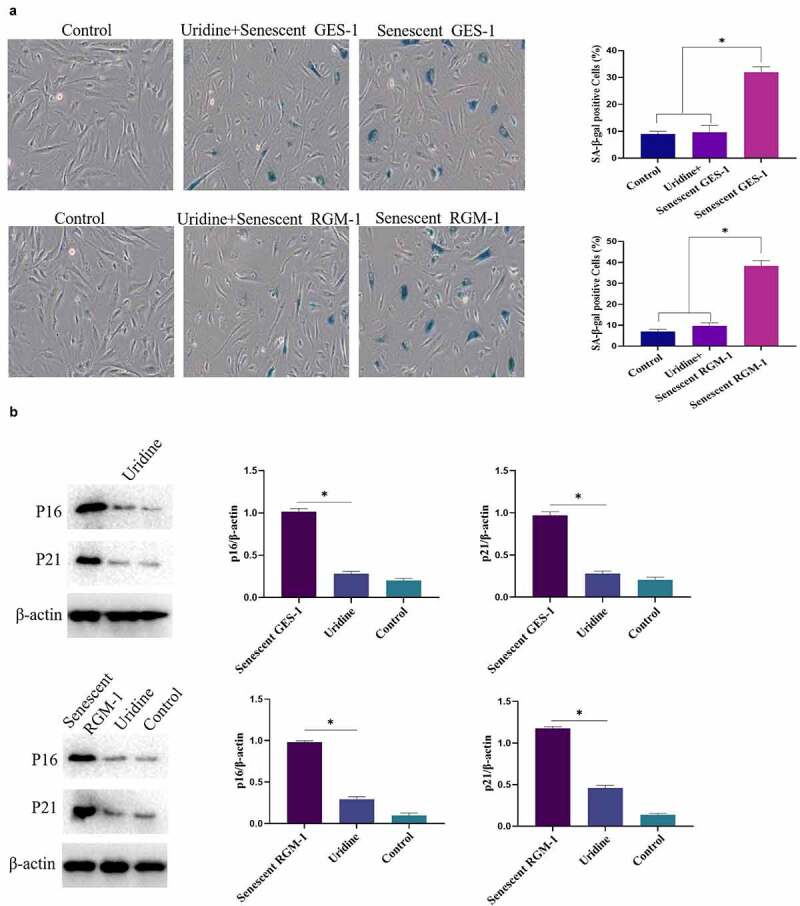
(a). the senescence-associated β-galactosidase (SA β-gal)-positive cell ratio were significantly reduced by uridine treatment. (b). The senescence-related markers (p16, p21 and p53) were down-regulated by uridine treatment. (c). MTT assay indicated that the uridine treatment increased cell proliferation ability of the senescent GES-1 and RGM-1 cell. (d). uridine treatment effected the cell cycle. The data are shown as means ± SEM. Asterisks indicate significant differences (P < 0.05).

### The effect of uridine on inflammation

**Figure 8.**
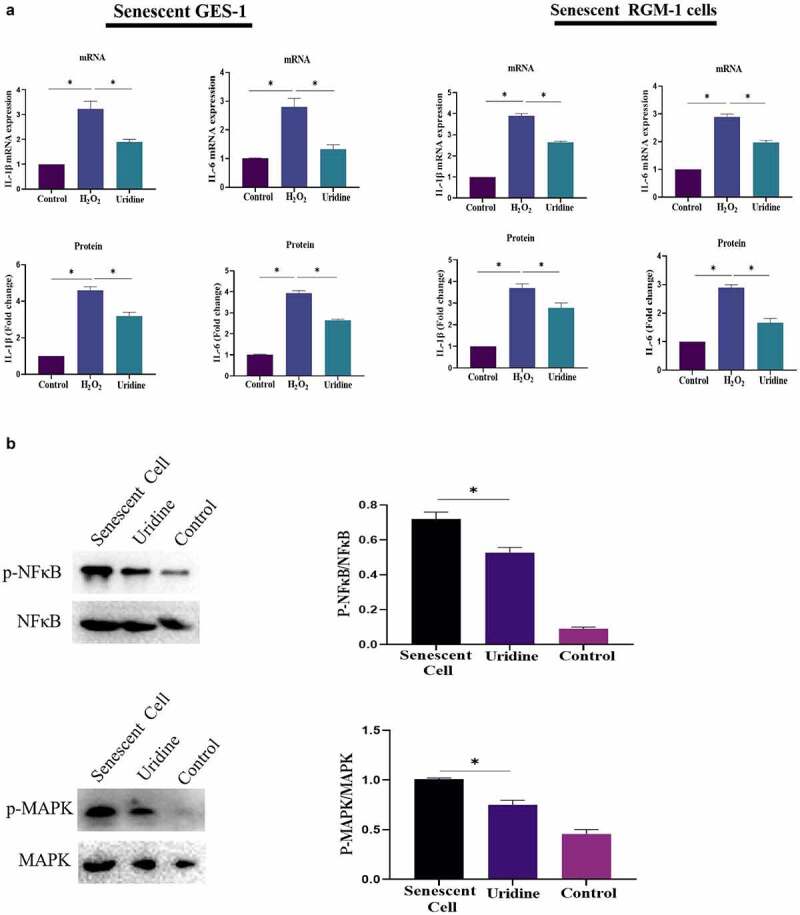
(a). Uridine treatment reduced the expression of IL-6 and IL-1β. The senescent GES-1 and RGM-1 cells were pre-treated with uridine (0.4 mg) for 24 h. The level of proinflammatory cytokines were analyzed by ELISA kit (Thermo, 88–50,625 for IL-6; Thermo, 88–7340 for TNFα and Thermo, 88–6010 for IL-1β) according to the manufacturer’s instructions. (b). Uridine pre-treatment inhibited inflammatory-related signaling pathway. The senescent GES-1 and RGM-1 cells were pre-treated with uridine (0.4 mg) for 24 h. The activation of NF-κB and MAPK signaling pathway were analyzed by Western-blot. The data are shown as means ± SEM. Asterisks indicate significant differences (P < 0.05).

The effect of uridine on the proinflammatory cytokines was evaluated, and the results showed that uridine treatment reduced the expression of IL-6 and IL-1β (Figure 8a). Next, the potential mechanism by which uridine could inhibit inflammation was explored, the results showed that uridine pre-treatment inhibited inflammatory-related signaling pathway (e.g. MAPK/NF-κB) (Figure 8b).

### The effect of uridine on oxidative stress

**Figure 9.**
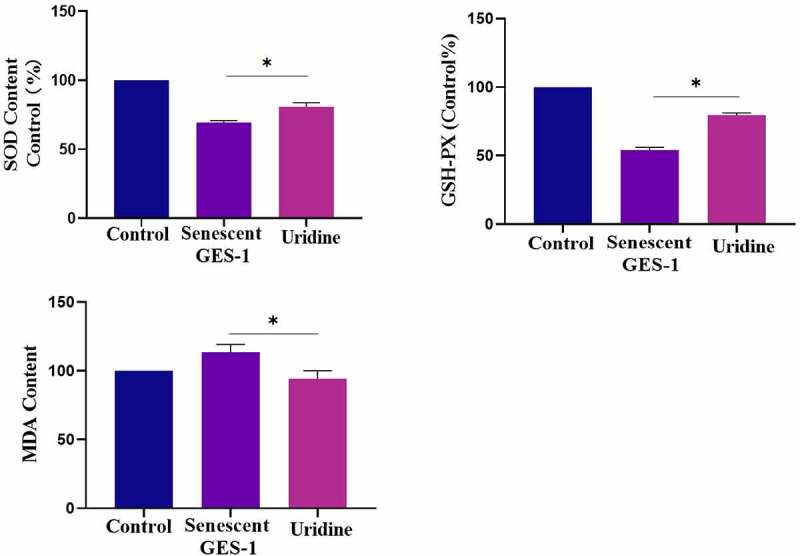
Uridine pre-treament attenuated the oxidative stress of the senescent GES-1 and RGM-1. The senescent GES-1 and RGM-1 cells were pre-treated with uridine (0.4 mg) for 24 h. Oxidative stress markers and anti-oxidative stress markers were determined as described in the materials and methods section. The data are shown as means ± SEM. Asterisks indicate significant differences (P < 0.05).

Aging is closely related to oxidative stress, and H_2_O_2_ treatment also may lead to oxidative stress. For this, we investigated the effect of uridine on oxidative stress. It can be found that the oxidative stress level was up-regulated in the senescent GES-1 and RGM-1 cells. However, uridine pre-treament attenuated the oxidative stress of the senescent GES-1 and RGM-1 as determined by oxidative stress markers and anti-oxidative stress markers (e.g. ROS, SOD) (Figure 9), these finings suggested that uridine shows the anti-oxidative stress effect.

## Discussion

The human digestive system is divided into digestive tract and digestive gland [14]. The digestive tract is mainly composed of stomach and small intestine. The elderly are experiencing the process of aging [15–19]. With the decline of physiological functions, the prevalence of some common gastrointestinal diseases has increased significantly in the elderly. Aging occurs in all biological organisms, which leads to tissue degradation and a variety of age-related diseases [20]. The physiological functions of the digestive system are mainly to ingest, transport, digest food and absorb nutrition, and finally excrete waste [14]. These physiological functions of digestive system play many important roles in regulating the physiological activities of the whole gastrointestinal tract. Gastrointestinal tract is closely related to human aging [21]. As the digestive system, the gastrointestinal tract can absorb nutrients and energy from the environment. The aging of gastrointestinal tract has contributed to the occurrence of age-related dysfunction [22]. Clinical studies have reported that the elderly is related to gastrointestinal dysfunction and gastric emptying disorders. When food enters into the intestine from the stomach, it needs to have a certain gastrointestinal motility, which is very important for the gastrointestinal tract to absorb nutrition and energy, and will also directly affect human health. Therefore, how to effectively alleviate the aging of gastrointestinal tract is an important scientific problem. In the current work, we studied the potential anti-aging effect of uridine, uridine is a precursor of pyrimidine nucleotides, which has many important bioactivites. In the current study, our study show for the first time that uridine has potential biological activity against aging. Uridine can alleviate aging by measuring a series of aging marker molecules (such as Sa-β-gal, p15 and p16). At present, anti-aging is a worldwide problem, therefore, uridine, as a potential anti-aging molecule, has its special advantages, it is hopeful to become an anti-aging functional food.

FGF has many important biological activities [23–26]. Previous studies have shown that FGF has important biological functions and roles, especially for the growth and development of stomach [27]. Therefore, in the current study, we also studied the effect of gastric cell aging on the biological activity of FGF. We firstly studied the cellular characteristics of FGF and found that gastric epithelial cells express abundant FGFR, and FGF could internalize into cells in a time-dependent manner. Interestingly, we found that FGF and FGFR could also transport into the nucleus. This phenomenon has been reported in tumor cells [28]. Studies showed that nuclear FGFR may be involved in cell proliferation [29]. However, the biological functions and roles of nuclear-localized FGF/FGFR in somatic cells has not been completely revealed, which needs further experiments to uncover in the future. In the present work, we found an interesting phenomenon that aging caused changes in the biological characteristics of FGF. The bFGF’s nuclear localization was inhibited in the senescent GES-1 and RGM-1, but its potential mechanism has not been fully revealed.

Next, we analyzed the effect of aging on the biological activity of FGF and found that FGF/ FGFR-mediated signaling pathway was significantly down-regulated in the senescent GES-1 and RGM-1cells, which suggests that aging seriously affected the bioactivities of FGF. Further, we explored the potential mechanism by which aging caused the down-regulation of FGFR-mediated signaling. We found that the expression pattern of FGFR was changed significantly, membrane -localized FGFR decreased in the senescent GES-1 and RGM-1 cells, but there was no significant change in the expression of total FGFR. However, the potential biological mechanism that membrane-localized FGFR was down-regulated has not been revealed, further work is needed to reveal this issue. In addition, our study found that uridine could partially alleviate the down-regulation of FGF signaling. However, the specific mechanism needs further research in the future.

## Conclusions

In the current study, we established the senescent cell model *in vitro*, and further study indicated that the biological activity of FGF is down-regulated in the senescent cell model. Furthermore, we found that uridine has anti-aging potential and can partially restore the sensitivity of FGF. In short, current work indicates that: 1) uridine can alleviate the senescence of gastric cells; 2) The senescence of gastric cell reduces the biological activates of FGF; 3) Uridine can partially restore the sensitivity of gastric cell to FGF.

## Data Availability

All data can be obtained from the corresponding author upon reasonable request.

## Limitation/short-coming of the study

The potential molecular mechanism by which uridine inhibits cell aging needs further study in the future.

## Future direction

To analyze and study the anti-aging effect of uridine in vivo is the future research direction.
